# Autologous fat grafting: Latest insights

**DOI:** 10.1016/j.amsu.2018.10.016

**Published:** 2018-10-16

**Authors:** Maarten Doornaert, Julien Colle, Elisabeth De Maere, Heidi Declercq, Phillip Blondeel

**Affiliations:** University Hospital Gent, Belgium

**Keywords:** Fat grafting, ASC, Lipofilling, Lipotransfer, White adipose tissue engineering, CAL, Cell

## Abstract

A recent rise in the use of autologous fat transfer for soft tissue augmentation has paralleled the increasing popularity of liposuction body contouring. This creates a readily available and inexpensive product for lipografting, which is the application of lipoaspirated material. Consistent scientific proof about the long-term viability of the transferred fat is not available. Clinically, there is a reabsorption rate which has been reported to range from 20 to 90%. Results can be unpredictable with overcorrection and regular need for additional interventions. In this review, adipogenesis physiology and the adipogenic cascade from adipose-derived stem cells to adult adipocytes is extensively described to determine various procedures involved in the fat grafting technique. Variables in structure and physiology, adipose tissue harvesting- and processing techniques, and the preservation of fat grafts are taken into account to collect reproducible scientific data to establish standard in vitro and in vivo models for experimental fat grafting. Adequate histological staining for fat tissue, immunohistochemistry and viability assays should be universally used in experiments to be able to produce comparative results. By analysis of the applied methods and comparison to similar experiments, a conclusion concerning the ideal technique to improve clinical outcome is proposed.

## Highlights

• Adipogenic physiology is described to determine various procedures involved in the fat grafting technique.
• Clinical studies on fat grafting have confirmed an unpredictable result.
• After analysis of the literature and despite attempts to eliminate confounding factors, on every step of the fat transfer technique a number of studies with conflicting results exist.
• Adequate histological staining for fat tissue, immunohistochemistry and viability assays should be universally used in experiments to be able to produce comparative results.

## 1 White adipose tissue

According to the World Health Organization, the incidence of obesity has tripled since 1975. This has incited an increasing demand for liposuction and contour surgery during these last decades. Concurrently, our views and understanding of fat tissue have changed. Fat has evolved from a waste tissue impeding the way to the important surgical sites, to an important and potential source of cells for reconstructive medicine.

White adipose tissue was originally considered a fairly inert energy storage tissue consisting of a fixed number of adipocytes. However, adipose tissue continuously varies in size throughout life. Adipocytes gain size during lipid accumulation, but recent advances in adipose biology have demonstrated that an increase in adipocyte size often is followed by an increase in adipocyte numbers [[1], [2], [3], [4]]. Adipocytes derive from multipotent mesenchymal stem cells, now conventionally called adipose-derived stem cells (ASCs). ASCs can both proliferate or differentiate into adipocytes. In this process, numerous intermediate cell types exist, difficult to characterize. For practical reasons, two obvious phases in adipogenesis are most frequently described. In the first or determination phase, a stem cell is committed to the adipocyte lineage, and thus called pre-adipocyte. To accomplish this, a growth arrest is required, which is normally achieved through contact inhibition. No morphological difference can be made between the pre-adipocyte and its precursor, but the cell has lost its potential to differentiate into other cell types. In the second or terminal differentiation phase, the pre-adipocyte takes on the characteristics of the mature adipocyte, with lipid accumulation in the cytosol displacing the nucleus from the center to the periphery of the cell. Late markers of differentiation, such as glycerol-3-phosphate dehydrogenase (G3PDH) and fatty acid synthetase (FAS) are now detectable [5].

During this adipogenic cascade, the signal transduction pathway is regulated by a large number of hormones, cytokines and growth factors. Insulin, IGF-1, glucocorticoids are among the positive effectors, while cytokines, TGF-β family growth factors and protein kinase C (PKC) inhibitors are viewed as negative regulators. On the transcriptional level, key regulatory events include the induction of CCAAT/enhancer binding proteins (C/ΕΒPs), but the master role is played by peroxisome proliferator-activated receptor-y (PPAR-y). No other factor has been discovered that promotes adipogenesis in the absence of PPAR-y, while it is on itself sufficient for adipogenesis [6].

Mature adipocytes synthesize proteins involved in lipid and steroid metabolism. Leptin, a well-known example, plays a crucial role in the regulation of energy balance and its levels are increased in obesity. It has also been found to increase the vascular permeability in adipose tissue, and consequently influence the microvessel density [7]. Tumor necrosis factor-α (TNF-α), interleukin (IL) −6 and −8 are pro-inflammatory proteins that are increasingly synthesized by adipocytes in obesity and play a role in insulin resistance and lipolysis. In vitro, adipocytes from newly cultured explants of human subcutaneous adipose tissue rapidly express TNF-α and downregulate PPAR-y in vitro as a catabolic response, even after most gentle tissue handling [8].

The basic organization of a white fat depot consists of mature adipocytes, stromal-vascular cells, blood vessels, lymph nodes and nerves. The stromal vascular cell (SVF) fraction contains ASCs, pre-adipocytes, endothelial cells, pericytes, macrophages and fibroblasts. The phenotype of the ASCs in the SVF was described in a conjoint statement of the International Federation for Adipose Therapeutics (IFATS) and the International Society for Cellular Therapy (ISCT) in 2013 as CD34 ^+^ CD45^−^CD31^−^CD273a-CD73 ^+^ CD13^+^ [9]. ASCs resemble the type of mesenchymal stem cells, that, since their original description in the 1960s [10,11], have been found in nearly all adult tissues. The exact location of the stem cell populations is suggested to be in the perivascular niche [12].

Adipose tissue is highly vascularized, and it is postulated that each adipocyte is in close proximity to a blood capillary allowing for efficient exchange of metabolic products. As adipose tissue continuously undergoes expansion and regression throughout adult life, it requires the parallel growth of its capillary network. ASCs can release multiple angiogenesis-related growth factors including Vascular Endothelial Growth Factor (VEGF) and Hepatocyte Growth Factor (HGF) [13] and have shown to trigger blood vessel formation in collagen gels in vitro [14]. On the other hand, endothelial cells sustain pre-adipocyte viability, proliferation and adherence when subjected to defined hypoxic conditions [15]. Clinically, the revascularization capacities of the fatty omentum on bowels or when used in sternal reconstruction are well described. It is, amongst other reasons, the great synergistic potential between adipogenesis and angiogenesis in fat tissue that fuels the interest for using adipose tissue cells in tissue engineering.

## 2 Towards reconstructive medicine

An important feature for subcutaneous fat, is that it can easily be obtained by the minimally invasive procedure of liposuction. This procedure is well tolerated, safe, and low-cost. Adipose tissue contains a large number of mesenchymal stem cells, compared to bone marrow. A bone marrow transplant contains approximately 6 × 10^6^ nucleated cells per ml [16], of which only 0,001–0,01% are stem cells [17]. In comparison, subcutaneous liposuction provides approximately 0,5–2,0 × 10^6^ cells per gram of adipose tissue [16,[18], [19], [20], [21]], whereby the percentages of stem cells range from 1 to 10% [20,22,23]. While ASCs can be harvested from omental or visceral fat, the reconstructive potential does not appear to be different from subcutaneous fat, and harvesting would be more invasive [24].

## 3 *In vitro* potential

**Fig. 1:**
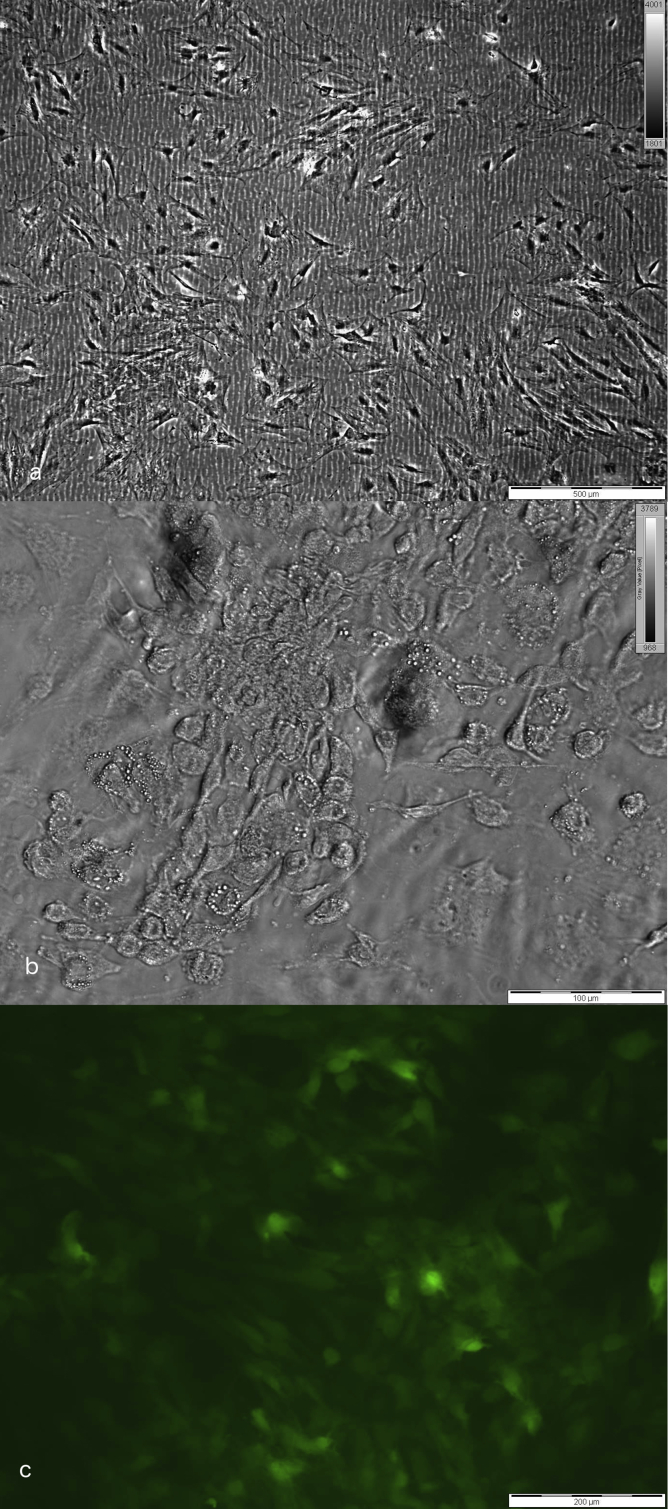
a: typical ASC culture. Cells are spindle shaped, and rapidly expanding. b: culture in adipogenic medium results in a more rounded shape and accumulation of lipid droplets in the cytoplasma, characteristics of adipocytes. c: Lentiviral transduction of green fluorescent protein allows for easy tracking of ASC's in 3D gel constructs. (For interpretation of the references to colour in this figure legend, the reader is referred to the Web version of this article.)

The relative ease by which ASCs can be obtained has incited a large number of experiments on their reconstructive potential. Adipose SVF cells can be easily isolated from the lipo-aspirate with enzyme digestion, or, more recently, mechanical protocols. They are far more resilient to manipulation than mature adipocytes, and fairly easy to expand in a typical mono-layer culture, in standard media supplemented with fetal bovine serum. They can easily be expanded for multiple passages, until large numbers of cells are achieved (Fig. 1). Within the heterogeneous SVF cells, the subgroup of ASCs is usually isolated through plastic adherence in culture conditions.

Currently, cell therapeutic and tissue engineering experiments investigate the regenerative and therapeutic potential. Cultured ASCs have shown enormous differentiation potential in vitro. Logically, they can differentiate according to classic mesenchymal phenotype, into adipocytes, osteocytes and chondrocytes [[25], [26], [27]]. Interestingly, they also show potential for differentiation to neuron-like cells [[28], [29], [30]], epithelial cells [31], hepatocytes [32,33], pancreatic cells [34] and hematopoietic supporting cells [26,35,36].

Consequently, some researchers suggest that ASCs exert therapeutic potential through differentiation towards a specific cell type in the target tissue, while other, mostly in vivo animal studies contradict this [[37], [38], [39]]. They argue that despite the phenotypic differentiation of the ASCs into the target cell the full functionality is missing.

Most research has focused on the therapeutic potential that resides in the array of cytokines and growth factors secreted by ASCs, especially in hypoxic circumstances. These include angiogenic cytokines: Hepatic Growth Factor (HGF), Vascular Endothelial Growth Factor (VEGF), Fibroblast Growth Factor 2 (FGF-2), basic Fibroblast Growth Factor (b-FGF); Hematopoietic cytokines: Granulocyte-Colony Stimulating Factor (G-CSF), Granulocyte/Macrophage-Colony Stimulating Factor (GM-CSF), Interleukine-7 (IL-7), Monocyte-Colony Stimulating Factor (M-CSF); Pro-inflammatory cytokines: IL-6, IL-8, IL-11, TNF- [36]; anti-inflammatory cytokines: prostaglandin E2 [40]. A large number of studies confirms the ability of ASCs to promote tissue regeneration by secreting these growth factors, including the central nervous system, the heart, the kidneys, ischemic limbs and muscles, skin and scar tissue [[41], [42], [43], [44], [45], [46], [47], [48]]. Especially the angiogenic properties are impressive and well described [14,[49], [50], [51]].

Tissue engineering approaches focus more on biocompatible scaffolds and constructs incorporating the ASCs, differentiated or undifferentiated, alone or in combination with other cell types. Differentiated ASCs can replace the cells in the target tissue [52,53], while undifferentiated ASCs exert more diverse paracrine activity [54,55]. Besides the classic mesenchymal tissues such as bone [[56], [57], [58], [59]], cartilage [60], adipose tissue [61,62], studies have equally focused on skin tissue engineering [63] or nerve reconstruction [52,64]. Biocompatible materials for cell seeding include hyaluronic acid constructs, collagen type I, fibrin or polymers. The main problem to overcome is the vascularization of larger (solid) constructs at implantation in vivo. Vascularization requires organization of the constructed tissue. Different approaches to accomplish a vascular network have been suggested: applying mechanical stimulation, using biomaterials with appropriate properties, and microfabrication techniques such as 3D cell-printing. Others circumvent this issue by focusing on cell-laden injectable gel matrices, acting more like dispersed tissue grafts in the host tissue.

Finally, ASCs exhibit immunomodulatory properties, and have shown to protect against graft versus host disease after allogeneic stem cell transplantation [[65], [66], [67]]. According to the clinical trials database, 112 clinical studies are currently being performed using ASCs, including diabetic foot, crohn's disease, stroke, spinal cord injury and facial rejuvenation [68].

## 4 Clinical approach

**Fig. 2:**
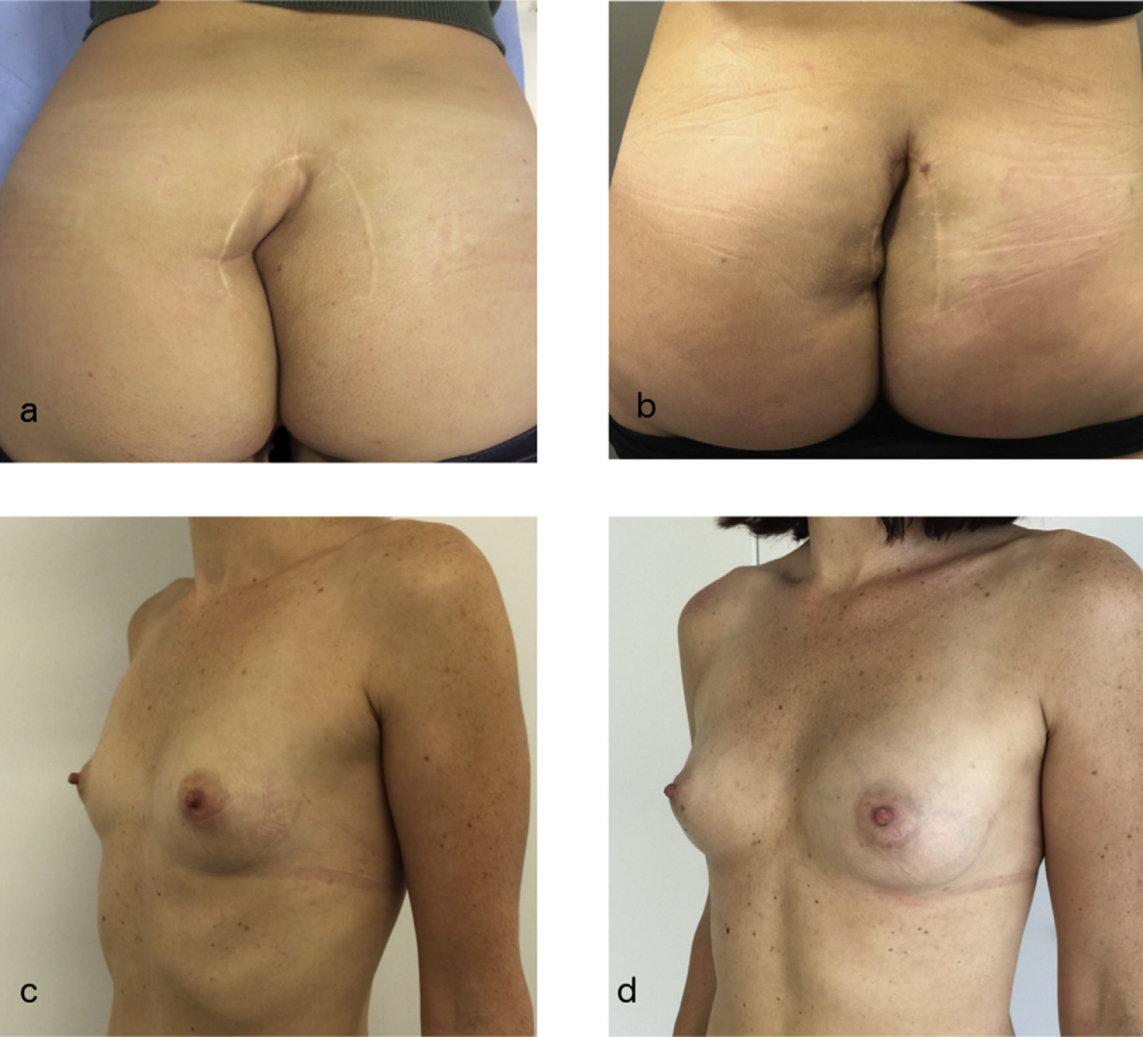
a: contour deformation and contracted scar after sacrococcygeal cyst removal at young age. b: restored contours after 2 sessions of lipofilling. c: mammary hypotrophy in a healthy young female patient. d: result after 1 session of lipofilling to the breast.

**Fig. 3:**
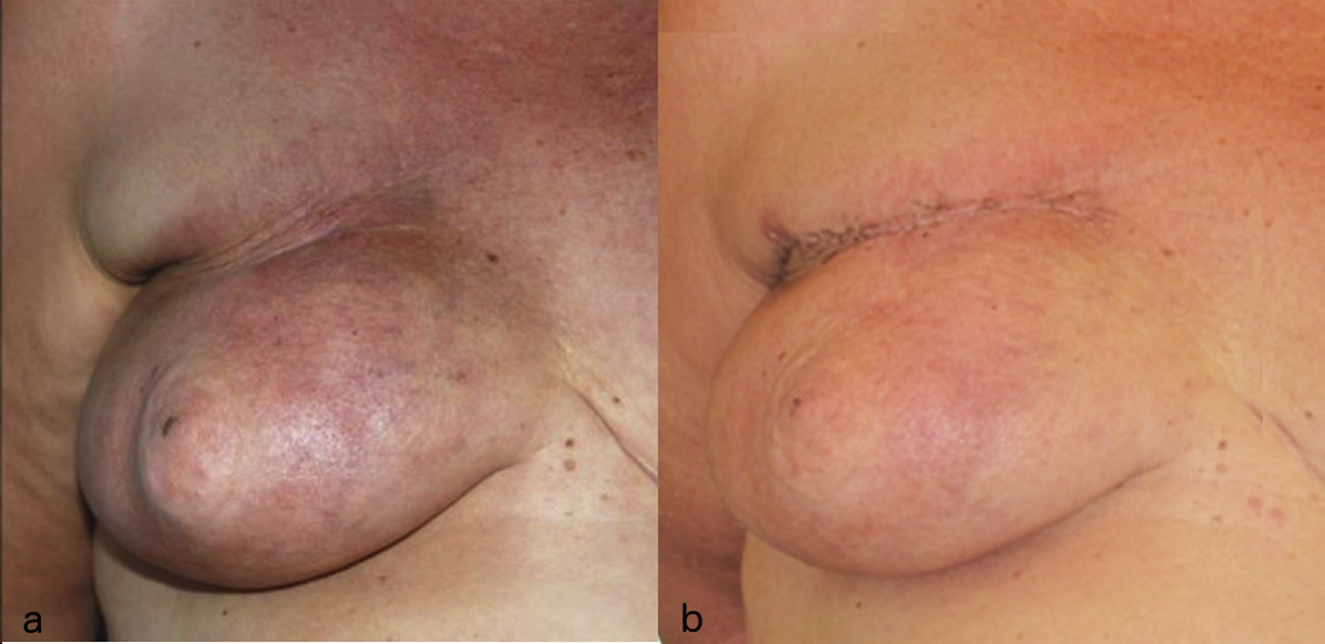
A: Status of a breast after tumorectomy and radiotherapy resulting in ischemic changes in the skin and retraction of the scar. B: Results after 2 sessions of lipofilling.

In the intra-operative setting of the current clinical practice, lipo-aspirate from liposuction is used on a daily basis. Indications include volume and contour restoration and scar treatment and release (Fig. 2), although tissue restoration is equally accepted (Fig. 3). The procedure includes harvesting, preparation of the lipo-aspirate, addition of substances, and reinsertion of the final product. Most harvesting procedures involve infiltration with Klein solution, now a generic name for physiologic saline solution containing a local anesthetic such as lidocaine, and epinephrine. Most researchers agree there is no serious negative effect of these substances on the fat cells [[69], [70], [71], [72]], while they reduce the risk of complications of the liposuction. Negative pressure liposuction with 3 or 4 mm diameter blunt cannula's is performed through stab incisions. For finer lipo-aspirate, cannula's of 1 mm can be used. A number of studies have underlined the importance of larger bore cannula's and low harvesting pressure for maintaining optimal adipocyte viability [[73], [74], [75], [76], [77], [78]]. Viable adipose cells can be successfully harvested from the abdomen, flanks, thighs, and medial knees, but there appears to be a higher yield of ASCs in fat harvested from the abdominal region and inner thighs [77,79,80]. Clinically, this might correlate with regions that expand more during excess caloric intake.

Next, during the lipo-aspirate preparation step, the lipo-aspirate is subjected to an intra-operative purifying procedure. Various techniques to extract the blood and oil from the lipo-aspirate are employed, varying from centrifugation at various speeds, to filtering through meshes, cleansing with various solutions, or combinations of these. Most authors agree on handling a fat graft as gently as possible, while at the same time allowing for removal of dead cells, oil, liposuction fluids and blood components [[81], [82], [83]] Finally, the processed fat graft is injected with blunt 1–2 mm cannulas, through small needle holes, in the area to be reconstructed.

Some authors advocate cell-assisted lipotransfer (CAL) [84,85]. In a one-stage procedure, a part of the fat tissue is processed for mechanical or enzyme-digesting SVF isolation, and these cells are then added to the rest of the adipose tissue for transplantation. Without more extensive manipulation of the SVF fraction, we can however not use the term ASCs for these cells. In a two-stage procedure, cell culture can expand the SVF fraction and isolate the ASCs through plastic adherence. Since GMP-level facilities and care is required, this adds costs to the procedure which can be as high as 10.000 € [86]. Based on clinical studies, no adequate level III or IV evidence can support the use of CAL [87,88].

Clinically, there is a reabsorption rate which has been reported to range from 20 to 90%. Results can hence be unpredictable with overcorrection on one hand and regular procedural repeats on the other. The lipotransfer technique involves a large number of variables that can influence the outcome of the graft, and therefore it is difficult to draw straightforward conclusions on appropriate methods from clinical studies. Recently, there has been a surge in experiments set up to recreate one part of the fat transfer process in a controlled setting. In this way each variable can be analyzed and determined for best outcome. However, as long as the fate of the cells composing the graft, or the influence of the recipient tissue is not fully elaborated, viability testing protocols after processing might not necessarily correlate with a good clinical outcome in vivo.

Therefore, a number of recent studies have focused on the fate of the cells composing the fat graft in vivo. Fat grafts initially require nutritional diffusion until vascularization from the recipient bed occurs. Histologically, in clinically failed grafts, progressive loss of adipocytes is noted along with a conversion of the graft in fibrous tissue and cysts [89]. Presumed mechanisms are primarily insufficient vascularity and inflammation around tissue debris. Therefore, theoretically, smaller fat deposits and particles are completely revascularized in a shorter time. Yet, this conflicts with the larger bore cannula's in harvesting being more beneficial to fat survival.

Undifferentiated preadipocytes, which are 20 times smaller than adipocytes, have a higher tolerance to ischemia than mature adipocytes [90]. Shear stress and mechanical trauma are prone to affect the larger and fragile lipid-laden adipocytes than the smaller and more resilient precursor cells.

Currently, two theories on the role of the different cells composing the fat graft after injection at the recipient site, stand opposed to each other.

The “graft survival theory”, first described by Peer et al. [91], states that the fat graft survives through imbibition until neo-vascularization from the recipient site occurs. This theory is adhered by those authors that advocate fat atraumatic processing of the fat graft to ensure the highest viability prior to injection [92].

In contrast, a “graft replacement theory” has gained importance, supported by a number of studies. Eto et al. [93] presented the outcome of their landmark in vivo mouse study on the three-zone survival theory in 2012. Inguinal fat pads were transplanted to the scalp area, and stained at 0, 1, 2, 3, 5, 7, or 14 days. They observed three zones from the periphery to the center of the graft: the surviving area (adipocytes survived), the regenerating area (adipocytes died, adipose-derived stromal cells survived, and dead adipocytes were replaced with new ones), and the necrotic area (both adipocytes and adipose-derived stromal cells died) [93]. It was thus concluded that very few adipocytes survive the grafting process and are replaced by newly differentiated ASCs co-transplanted in the graft.

These results were corroborated by Fu et al. [94], who found convincing evidence that the donor stromal vascular fraction cells participate in adipogenesis and angiogenesis.

However, others advocate a different replacement theory, stating that the cells replacing the necrotizing graft completely originate from recipient tissue. Neuhof and Hirshfeld [96] found that in the first months after transplantation, grafted cells necrotized and were gradually replaced by fibrous tissue and newly formed metaplastic fat both originating from recipient tissue. Dong et al. [95] corroborated these results with an elegant animal study. A cross-graft mouse model with transplantation of fragmented and integral inguinal fat pads was used and both angiogenesis and adipose retention in the graft were found to be recipient-dominated. These results support a “host cell replacement theory”, which states that no grafted cells survive, and all cells are replaced by cells from recipient origin. This would reduce the contribution of the fat graft to both spacing by mature adipocytes and providing paracrine stimuli by grafted ASCs.

While these in vivo animal models provide a valuable insight in the fate of the fat graft, it should be noted that minced inguinal fat-grafts could preserve the spatial matrix of the adipocytes, more than in lipo-aspirated fat graft used in the clinical settings. The co-transplanted spatial structure could induce a bias towards replacement theories by enlarging the graft volume.

Further refinement in the “graft replacement” mechanism, was provided by Hong et al. [97], who generated an animal model using two transgenic reporter mice expressing different fluorescent signals (DsRed and green fluorescent protein). Tracing experiments elucidated the dynamic changes of donor ASC, donor fat and recipient tissue. They found that surviving donor ASCs participated in angiogenesis by differentiating into endothelial cells and described newly differentiated fat from donor ASC & recipient tissue integrated with surviving donor fat. A combination of graft replacement, survival and host replacement theories is thus supported.

Based on current research, it seems feasible that the eventual fat graft mechanism depends on all above-described theories. Gently processed adipocytes, deposited in small particles in proximity to recipient vascularization, survive the transplantation process. Recipient cells can be attracted by chemotaxis and equally contribute to structural and paracrine support in the graft, particularly in well-vascularized recipient sites. Co-transplanted stromal vascular fraction cells contribute in paracrine stimulation of vascularization and wound healing phases and provide structural support by differentiating into endothelial cells and adipocytes. In less vascularized recipient sites, the supplementation of ASCs in the graft could theoretically augment results. Future research will undoubtedly unravel the contribution of all above-describe theories.

In conclusion, a very promising area of research in adipose reconstructive medicine is developing. Experimental research focusing on ASC culture, expansion and tissue engineering constructs is converging with clinical reconstructive procedures, and the often-abundant energy reservoir that adipose tissue is regarded upon, might in the future prove to be the most useful repair tissue reservoir.

## Provenance and peer review

Not commissioned, externally peer reviewed.

## Ethical approval

N/A.

## Sources of funding

The authors received no specific funding for this work.

## Conflicts of interest

We have no conflict of interest to declare.

## Consent

N/A.

## Author contribution

Dr. Maarten Doornaert: study design, data collection, data analysis and interpretation. Writing the paper.

Dr. Julien Colle: Data interpretation, reviewing the article.

Miss. Elisabeth De Maere: Data interpretation, reviewing the article.

Dr. Heidi De Clercq: Data interpretation, providing cell culture pictures and advice

Prof. Phillip Blondeel: Reviewing the article, providing clinical relevant pictures.

## Research registration number

N/A.

## Guarantor

Dr. Maarten Doomaert.
